# Isolation and characterization of two virulent Aeromonads associated with haemorrhagic septicaemia and tail-rot disease in farmed climbing perch *Anabas testudineus*

**DOI:** 10.1038/s41598-021-84997-x

**Published:** 2021-03-12

**Authors:** Abhishek Mazumder, Hrishikesh Choudhury, Abhinit Dey, Dandadhar Sarma

**Affiliations:** Department of Zoology, Gauhati University, Guwahati, Assam 781014 India

**Keywords:** Bacterial pathogenesis, Bacteriology, Infectious-disease diagnostics

## Abstract

Diseased *Anabas testudineus* exhibiting signs of tail-rot and ulcerations on body were collected from a fish farm in Assam, India during the winter season (November 2018 to January 2019). Swabs from the infected body parts were streaked on sterilized nutrient agar. Two dominant bacterial colonies were obtained, which were then isolated and labelled as AM-31 and AM-05. Standard biochemical characterisation and 16S rRNA and *rpo*B gene sequencing identified AM-31 isolate as *Aeromonas hydrophila* and AM-05 as *Aeromonas jandaei*. Symptoms similar to that of natural infection were observed on re-infecting both bacteria to disease-free *A. testudineus*, which confirmed their virulence. LC_50_ was determined at 1.3 × 10^4^ (*A. hydrophila*) and 2.5 × 10^4^ (*A. jandaei*) CFU per fish in intraperitoneal injection. Further, PCR amplification of specific genes responsible for virulence (aerolysin and enterotoxin) confirmed pathogenicity of both bacteria. Histopathology of kidney and liver in the experimentally-infected fishes revealed haemorrhage, tubular degeneration and vacuolation*.* Antibiotic profiles were also assessed for both bacteria. To the best of our knowledge, the present work is a first report on the mortality of farmed climbing perch naturally-infected by *A. hydrophila* as well as *A. jandaei*, with no records of pathogenicity of the latter in this fish.

## Introduction

*Aeromonas* is a genus comprising of Gram-negative, oxidase-positive, non-spore-forming, facultative, anaerobic, rod-shaped bacteria responsible for causing infectious diseases in fishes as well as humans^1,2^. With a high degree of adaptability in fresh, estuarine as well as marine habitats, these microorganisms are one of the major etiological agents of fish species and other aquatic organisms^3^. The mesophilic bacterium *Aeromonas hydrophila* is an opportunistic and zoonotically-important motile fish pathogen reportedly responsible for causing epizootic ulcerative syndrome, haemorrhagic septicaemia and pathological changes on the tail and fins of fishes^4,5^. Toxins identified in *A. hydrophila*, viz. haemolysins, enterotoxins and/or endotoxins, are the major virulence factors that lead to their pathogenicity and overall disease progression^6^. Freshwater teleosts like channel catfish, pacu, tilapia and trout, when infected with *A. hydrophila*, show poor growth, loss of appetite, erratic swimming, stagnation and anorexia, and free mucus and ulceration on fins and/or body, respectively^4,5,7–10^. Pathogenicity of *Aeromonas jandaei* is reported in a few fish species like *Anguilla anguilla*^11^, *Pangasianodon hypophthalmus*^12^ and *Oreochromis niloticus*^13^. However, *A. jandaei* is also known to cause wound infections in immunocompetent and immunocompromised humans when exposed to freshwater sources^14,15^.

The freshwater air-breathing teleost, *Anabas testudineus* is a highly demanded food fish in India due to its nutrient-rich flesh. Being ubiquitous throughout Indochina and Southeast Asia^16^, *A. testudineus* has the potential to be a suitable candidate species of fish farming. However, farmed *Anabas* are prone to bacterial infections mostly due to the presence of *Aeromonas*, *Flavobacterium*, *Pseudomonas*, *Salmonella* and *Staphylococcus*^17^. Apart from being unfit for consumption, a diseased fish exhibiting ulcerations and tail-rot becomes aesthetically unpleasant to the consumer. This can eventually lead to a lesser demand for the species in the local fish markets, and, consequently, create huge economic loss to the fish farmer. During the winter season of 2018–2019 (i.e. between November and January), ulceration and fin-rot followed by high mortality (approximately 50%) was recorded in individuals of *A. testudineus* in a local fish farm of Assam, India. The primary objective of the present study was to isolate and characterize the (predominant) bacteria causing mortality in these diseased *Anabas* individuals, and confirm the pathogenicity of the isolated bacteria via search of specific virulence-associated genes and experimental re-infections in disease-free *A. testudineus*.

## Results

### Morphological and biochemical characterization

Dominant colonies were obtained of the two bacterial isolates, AM-31 and AM-05, which displayed circular morphology with average diameter of 2–3 mm and appeared yellowish on nutrient agar. Both isolates were motile, Gram-negative, oxidase positive, catalase positive, O/129 resistant and produced gas on glucose fermentation. All tested biochemical characteristics of both isolates are presented in Table 1.

**Table 1:** Selected biochemical characteristics of two bacterial isolates (AM-31 and AM-05) retrieved from swabs of infected body parts and kidney of naturally-infected *Anabas testudineus* collected from a fish farm of Assam, India (*ND* not determined, + positive, − negative).

Characters	Characterization of *A. hydrophila* after Popoff and Véron^43^	Isolate AM-31	Characterization of *A. jandaei* after Abbott et al.^44^	Isolate AM-05
Gram stain	−	−	−	−
Shape	Rod	Rod	Rod	Rod
Motility	+	+	+	+
Oxidase	+	+	ND	+
Catalase	+	+	+	+
Urea	−	−	ND	−
Acid and gas production from glucose	+	+	+	+
**Acid production**				
Lactose	+	+	+	+
Sucrose	+	+	−	−
Maltose	+	+	−	+
Mannitol	−	−	+	+
Inositol	−	−	−	−
Sorbitol	−	−	−	−
Esculin hydrolysis	+	+	+	−
Starch hydrolysis	ND	+	ND	+
Methyl-red test	−	−	+	+
Voges-proskaur	+	+	+	+
Indole	+	+	+	+
H_2_S production	+	+	+	+
**Growth at**				
4 °C	−	−	ND	−
5 °C	+	+	ND	+
37 °C	+	+	ND	+
40 °C	−	−	ND	−
**Salt tolerance (% NaCl)**				
0%	+	+	+	+
3%	+	+	+	+
6%	ND	−	−	−
Citrate utilization	+	+	+	+
Arginine decomposition	+	+	+	+
Lysine decarboxylation	−	−	+	+
Ornithine decarboxylation	−	−	−	−

### Molecular identification based on 16S rRNA and *rpo*B genes

PCR amplification of total genomic DNA of both isolates using 16S rRNA bacterial universal primers yielded ~ 1500 bp amplicons. The results of 16S rRNA gene sequencing of isolate AM-31 revealed 99.93% similarity with four reference strains, viz. *A. hydrophila* ATCC 7966 (GenBank accession no. NR 074,841.1), *A. hydrophila* JCM 1027 (GenBank accession no. NA 113342.1), *A. hydrophila* ATCC 7966 (GenBank accession no. 118944.1), and *A. hydrophila* CCM 7232 (GenBank accession no.NR 043638.1); while that of the isolate AM-05 revealed 99.86% similarity with two reference strains, viz. *A. jandaei* CDCO787-80 (GenBank accession no.: NR 0370132) and *A. jandaei* ATCC 49568 (GenBank accession no.: NR 119040), respectively. Also, PCR amplification of the *rpo*B gene yielded amplicons of ~ 550 bp of both isolates. Isolate AM-31 had 100% query coverage with 99.40% identity with the reference strain *A. hydrophila* JCM 1027 (GenBank accession no.: LC200411), and isolate AM-05 had 99.61% identity with *A. jandaei* strain 1459 (GenBank accession no.: MG098879). An almost-complete 16S rRNA and *rpo*B sequence containing < 1% undetermined positions was obtained for both the isolates.

**Figure 1:**
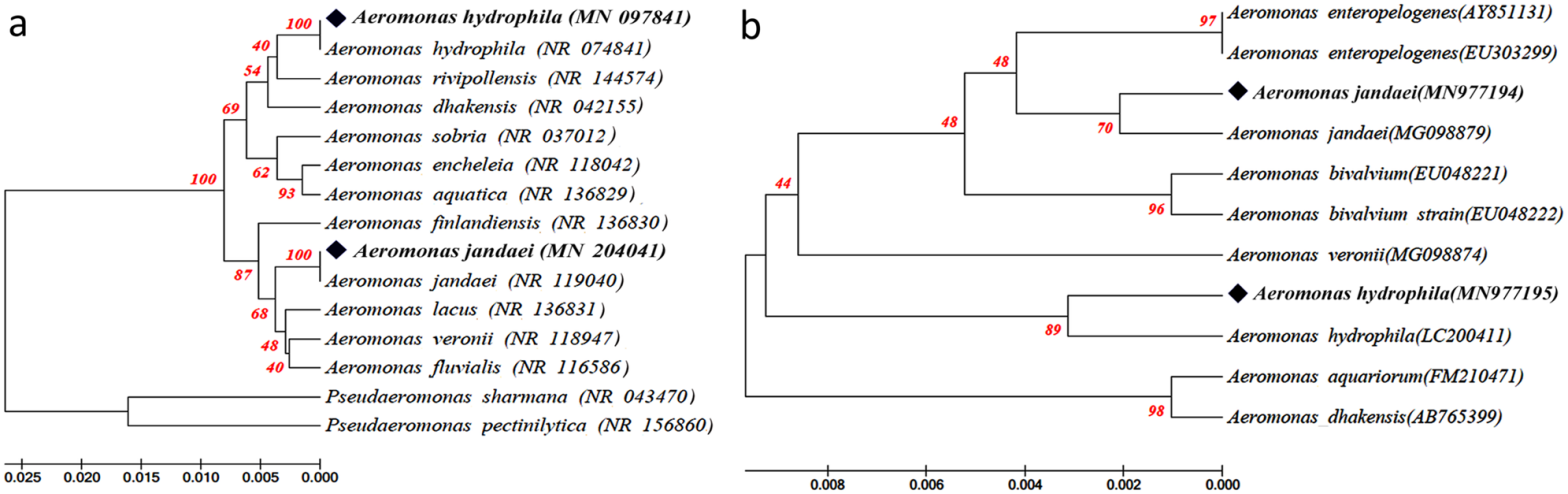
Phylogenetic tree (UPGMA model) based on 16S rDNA (**a**) and *rpo*B (**b**) of two bacterial isolates (AM-31: *Aeromonas hydrophila* and AM-05: *A. jandaei*) and their closely-related species. *Pseudomonas sharmana* and *P. pectinilytica* were selected as outgroups for the 16S gene. Percentage bootstrap values (1000 replicates) are shown at each branch point.

The gene sequences determined for the two representative isolates in this study were deposited in GenBank (Accession numbers: MN097841 and MN204041 for 16S rRNA gene, and MN977195 and MN977194 for *rpo*B gene). The phylogenetic tree (Fig. 1) clustered the isolate AM-31 with *Aeromonas hydrophila* and isolate AM-05 with *A. jandaei*, respectively. These clusters were also strongly supported by their high bootstrap values.

### Clinical signs and virulence

**Figure 2:**
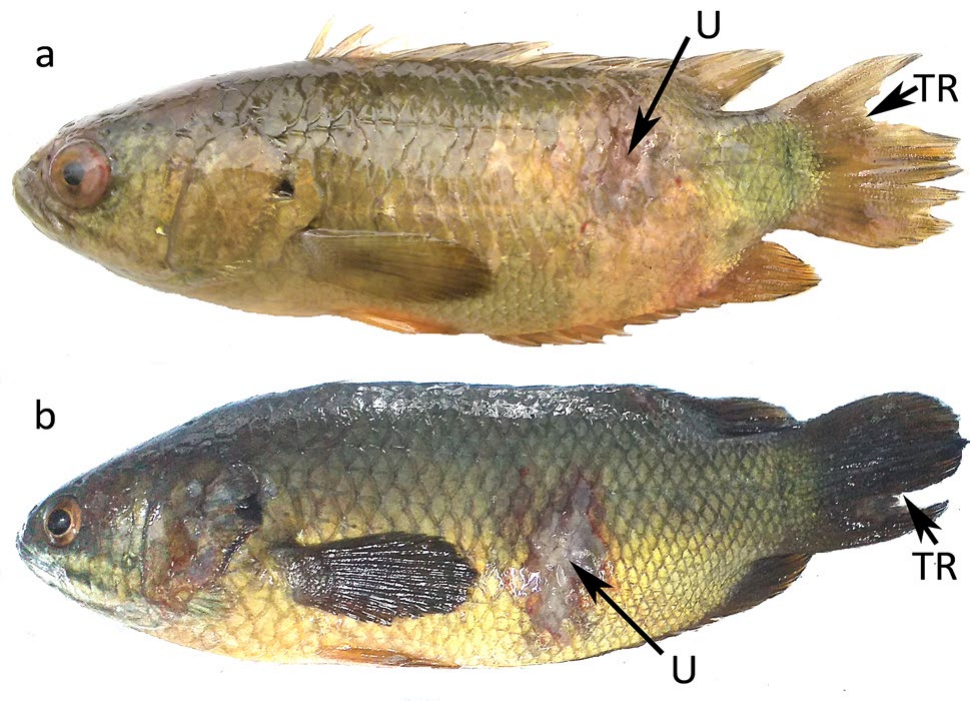
Tail-rot (TR) and Ulcerations (U) on body of *Anabas testudineus* experimentally-challenged with bacteria (**a**) *Aeromonas hydrophila* (AM-31) and (**b**) *A. jandaei* (AM-05).

Intraperitoneal injection of AM-31 and AM-05 isolates in disease-free *Anabas testudineus* individuals resulted in ulcerations and fin-rot, similar to as observed in natural infections (Fig. 2). The fishes developed symptoms of excessive mucus secretion, followed by gradual development of grayish-white lesions towards the posterior half of body that later extended to the caudal fin. The anal region exhibited scale loss, which then developed into ulcers, and the fins appeared reddish that later formed conspicuous fin rot. All clinical signs commenced within 48 h of artificial infection.

**Figure 3:**
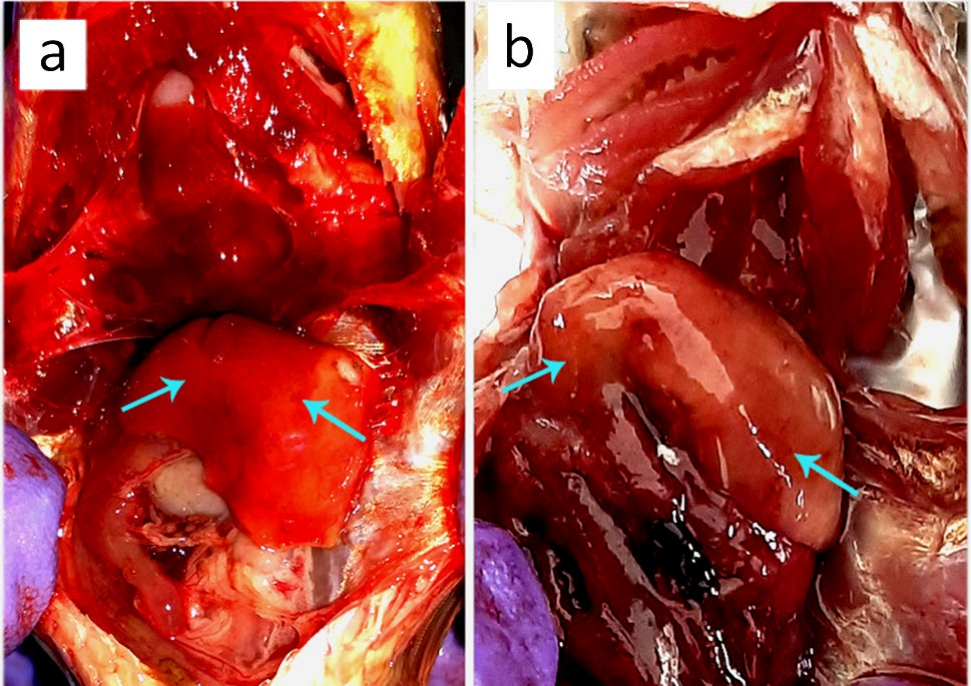
Haemorrhagic spots (blue arrows) on the surface of liver of *Anabas testudineus* experimentally-challenged with (**a**) *Aeromonas hydrophila* and (**b**) *A. jandaei*.

Mortality was recorded within 15 days, and the calculated lethal concentrations (LC_50_) for AM-31 and AM-05 were 1.3 × 10^4^ and 2.5 × 10^4^ CFU/fish, respectively (Table 2). The infected fish showed severe haemorrhage and blood congestions notably in the liver (Fig. 3). However, no mortality was recorded in the control groups (i.e. the PBS-injected and without injection).

**Table 2:** Results of *Aeromonas hydrophila* (isolate AM-31) and *A. jandaei* (isolate AM-05)-induced pathogenicity in experimentally-challenged *Anabas testudineus* via intraperitoneal injection showing mortality within 15 days of infection.

Injected with	Dose (CFU/fish)	No. of fish challenged	No. of fish died	Mortality (%)	LC_50_ value
*Aeromonas hydrophila*	3.4 × 10^2^	10	2	20	1.3 × 10^4^
	3.4 × 10^3^	10	4	40	
	3.4 × 10^4^	10	5	50	
	3.4 × 10^5^	10	8	80	
	3.4 × 10^6^	10	10	100	
	3.4 × 10^7^	10	10	100	
Control	0.1 ml PBS	10	0	0	
Control	Without injection	10	0	0	
*Aeromonas jandaei*	4.2 × 10^2^	10	1	10	2.5 × 10^4^
	4.2 × 10^3^	10	2	20	
	4.2 × 10^4^	10	5	50	
	4.2 × 10^5^	10	7	70	
	4.2 × 10^6^	10	10	100	
	4.2 × 10^7^	10	10	100	
Control	0.1 ml PBS	10	0	0	
Control	Without injection	10	0	0

### Hemolysin assay

Positive haemolytic activity (yellow colour) was observed for both isolates with halo diameters of 0.9 mm (AM-31) and 1.4 mm (AM-05), respectively.

### PCR detection of virulence-associated genes

Cytotoxic enterotoxin was detected in both the isolates. But, aerolysin gene was detected only in isolate AM-05.

### Histological changes in kidney and liver

**Figure 4:**
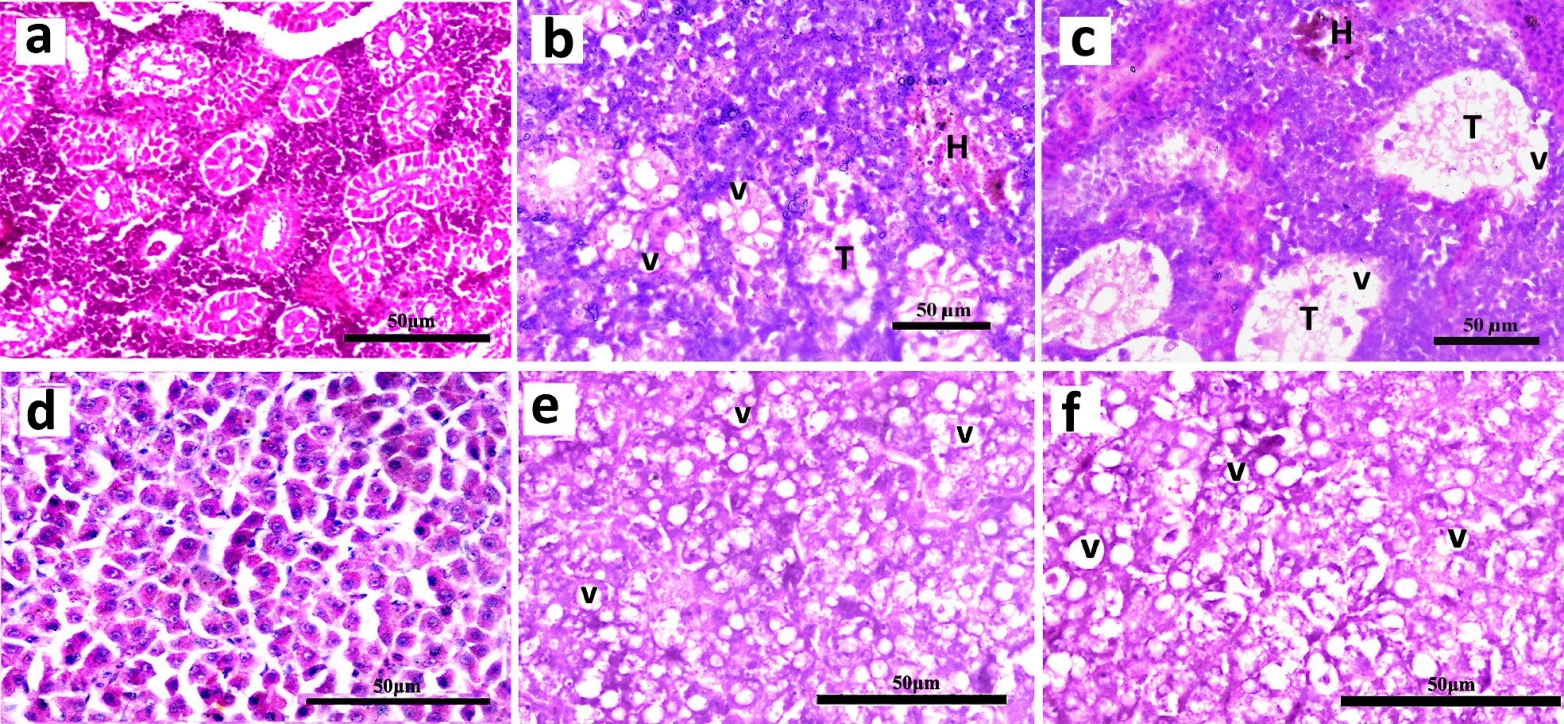
T. S. of (**a**) kidney and (**d**) liver in disease-free (as control) *Anabas testudineus*. T. S. of kidney of *A. testudineus* experimentally-challenged with (**b**) *Aeromonas hydrophila* and (**c**) *A. jandaei* [H, haemorrhage; T, vacuolar degeneration of renal tubules; V, vacuolation]. T. S. of liver experimentally-challenged with (**e**) *A. hydrophila* and (**f**) *A. jandaei* [V, vacuolation].

Histopathological examination of the experimentally-challenged *A. testudineus* showed haemorrhages and vacuolar degeneration of renal tubules of kidney and severe vacuolation in liver (Fig. 4).

### Sensitivity towards antimicrobials

AM-31 isolate showed sensitivity towards 15 of the 20 tested antibiotics (approximately 75%); whereas AM-05 was sensitive to all of them (i.e. 100%) (Table 3).

**Table 3:** Antibiotic susceptibilities interpreted as resistant (R) or sensitive (S) of two bacteria, *Aeromonas hydrophila* (AM-31) and *A. jandaei* (AM-05), isolated from naturally-infected *Anabas testudineus* [Zones of inhibition are expressed as mean (in mm) ± standard deviation (SD)].

Antimicrobial	Contents (µg)	AM-31		AM-05	
		Mean ± SD	Interpretation	Mean ± SD	Interpretation
Penicillin	10	9.3 ± 0.6	R	22.3 ± 0.3	S
Ampicillin	10	9.8 ± 0.2	R	19.2 ± 0.2	S
Ciprofloxacin	5	19.3 ± 0.7	S	20.0 ± 0.2	S
Chloramphenicol	30	19.0 ± 0.6	S	19.3 ± 0.2	S
Gentamycin	10	17.0 ± 0.2	S	18.1 ± 0.1	S
Erythromycin	15	18.5 ± 0.8	S	20.1 ± 0.1	S
Neomycin	10	16.0 ± 0.2	S	16.0 ± 1.9	S
Streptomycin	25	17.5 ± 0.5	S	21.7 ± 0.9	S
Imipenem	10	7.3 ± 0.3	R	17.3 ± 0.1	S
Kanamycin	30	19.0 ± 0.4	S	19.0 ± 0.4	S
Sulphonamide	300	17.7 ± 0.6	S	17.8 ± 0.9	S
Sulfisoxazole	300	18.8 ± 0.5	S	18.8 ± 0.5	S
Tetracycline	30	22.7 ± 0.4	S	22.7 ± 0.4	S
Enterofloxacin	5	18.0 ± 0.2	S	18.0 ± 0.2	S
Oxolinic acid	2	14.1 ± 0.2	S	14.1 ± 0.2	S
Deoxycycline	30	17.3 ± 0.1	S	17.3 ± 0.1	S
Fusic acid	10	7.2 ± 0.2	R	15.1 ± 0.1	S
Colistin	10	7.1 ± 0.2	R	12.3 ± 0.3	S
Florfenicol	30	18.2 ± 0.1	S	15.9 ± 0.5	S
Vancomycin	30	17.1 ± 0.1	S	19.2 ± 0.2	S

## Discussion

Based on the combination of biochemical and molecular characterization, the present study confirms the occurrence of *Aeromonas hydrophila* (isolate AM-31) and *A. jandaei* (isolate AM-05) in naturally-infected farmed *Anabas testudineus* with visible signs of tail-rot and ulceration on body.

Starch hydrolysis is one of the important tests to identify species of *Aeromonas*^18^. As *A. hydrophila* and *A. jandaei* both tested positive to starch hydrolysis (undetermined so far), this biochemical property will help in improved characterization and identification of the two species in future. Sorbitol fermentation and (lactose + urea) assimilation were found positive for both species, which is also an important phenotypic characteristic to distinguish mesophilic *A. hydrophila* from its complex members^19–21^. *A. hydrophila* differs from *A. jandaei* in characteristic of its sucrose and esculin hydrolysis positivity. Besides sucrose negative, *A. jandaei* is mannitol-positive; which further supports its earlier description as a genospecies DNA group 9 *A. sobria*, later re-named as *A. jandaei*^14^.

Molecular markers like 16S rRNA and *rpo*B have wide uses in identification, characterization and measurement of microbes. Sequencing of these two housekeeping genes has proven to be a useful tool in species delineation of the genus *Aeromonas*, and previous studies have successfully identified Aeromonads using the same^22–25^. Clustering of the bacterial 16S rRNA gene sequence of isolate AM-31 (GenBank Accession no.: MN097841), with maximum bootstrap values, confirmed its identity as *A. hydrophila*, placing *A. rivipollensis* and *A. dhakensis* as sister taxa. Similarly, the high sequence similarity of isolate AM-05 (GenBank Accession no.: MN204041) with referential sequences of *A. jandaei* confirmed its identity, placing *A. finlandensis*, *A. lacus*, *A. veronii* and *A. fluvialis* as sister taxa. The present molecular data validates the use of DNA sequencing in phylogenetic construction for enhancing our knowledge of bacterial identification and epidemiology^23^. The BLAST results of the *rpo*B fragments of both isolates AM-31 [99.4% similar to the reference strain of *A. hydrophila* (LC200411)] and AM-05 [99.6% similar to *A. jandaei* (MG098879)] additionally confirmed their identities. The phylograms based on both housekeeping genes, show consistent clustering of both isolates with their reference strains of *Aeromonas* species, thereby strongly supporting the present identification.

Virulence-associated factors in bacteria may include secreted enzymes^9^, cytotoxic enterotoxins^26,27^, hemolysins^28^, siderophores^29^ and aerolysins^30^, respectively. The positive response to haemolysin activity by *A. hydrophila* as well as *A. jandaei* in the present study confirms the toxicity of these isolates. Earlier studies on haemolytic activity of members of *Aeromonas*, too, reported toxicity to be strongly associated with enterotoxin production^31^, and that, haemolytic and cytotoxic activities could frequently relate a predominant bacteria to *A. hydrophila*^32^.

The pathogenicity tests were performed to analyse the capacity of *A. hydrophila* and *A. jandaei* to induce disease in healthy *A. testudineus*. The tests indicated their virulence to *Anabas* and the clinical signs of artificially-infected fish were similar to those observed in the naturally-infected individuals. Re-isolation of the same bacteria from experimentally-infected fish also fulfilled Koch’s postulates^33^. But, the nature of symptoms and the percentage mortality caused by the bacterial isolates at varied doses of intraperitoneal injection suggest that low concentrations of bacterial load (or with minimal stress factors) in the surviving environment leads to mild pathogenicity. Similar findings were reported in Nile tilapia re-challenged with *A. veronii* and *A. jandaei*^13^. Pathological lesions and mortality rate were milder in natural infection than in fishes infected artificially. Confinement of the fishes in aquaria during the experimental infection with increasing bacterial load on the host fish may have played a vital role to exhibit such symptoms.

Hossain et al.^34^ reported *A. hydrophila* to be pathogenic to *A. testudineus*, reporting 100% mortality @ 9.2 × 10^7^ CFU/fish. However, results obtained in the present experimental infection strongly proves *A. hydrophila* and *A. jandaei* to be equally pathogenic to *A. testudineus* with 100% mortality observed at much lower concentrations of 3.4 × 10^6^ and 4.2 × 10^6^ CFU/fish, respectively. The LC_50_ values for *A. hydrophila* (= 1.3 × 10^4^ CFU/fish) and *A. jandaei* (= 2.5 × 10^4^ CFU/fish) additionally support their lethality for farmed *Anabas* populations.

Screening for virulence-associated genes is one of the rational approaches in determining whether *Aeromonas* strains have the potential to be virulent^50^. Present study showed that *A. hydrophila* (isolate AM-31) and *A. jandaei* (isolate AM-05) were positive for the enterotoxin gene, which corroborates with the findings of Pablos et al.^35^. Further, this result is also concurrent with the haemolytic activity of these bacteria discussed earlier and, thus, both proving to be potent pathogens of *A. testudineus*. Contrary to the positive detection of aerolysin gene (aerA) in *A. jandaei*, *A. hydrophila* was, however, aerA negative. Although aerA gene is closely related to haemolytic, cytotoxic and enterotoxic activities of enterotoxin gene as reported in most *Aeromonas* strains^32^, the possible contradiction in the present study may be due to variations in prevalence of aerA in *Aeromonas* with respect to different geographical locations^36^.

Several studies reported that chronic infections of *A. hydrophila* cause dermal ulcerative lesions with focal hemorrhages and inflammation. Haemorrhage and vacuolar degeneration in renal tubules of kidney in experimentally-challenged specimens corroborates with similar findings made on *Oreochromis niloticus* experimentally-challenged with *A. hydrophila*^7^. Marked cytoplasmic vacuolations in liver tissues also correlate with previous studies on vacuolar degeneration in the parenchymatous organs and necrosis and haemorrhages caused by non-proliferative bacteria in teleosts^37^. These alterations may be toxin-induced owing to the presence of the enterotoxin gene in both bacterial isolates as discussed above.

AM-31 and AM-05 both, showed variable degrees of antibiogram resistance. Antibiotic resistance to penicillin and ampicillin as revealed for AM-31 isolate from the antibiogram study correlates with previous reports on multiple drug resistance of *A. hydrophila*^38^. However, *A. jandaei* was susceptible to all the antibiotics tested. Both bacteria exhibited high sensitivity for gentamycin and norfloxacin among all other antibiotics tested. Higher sensitivity for specific antibiotics (or the lack of any resistance towards them) may be due to the lack of awareness among local fish farmers within the Assam state (or northeast India) on the possible use of antibiotics towards disease prevention or treatment. Due to poor economic background, the farmers are more dependent on the traditional use of lime (calcium carbonate) and/or potassium permanganate as water disinfectant or disease treatment. Thus, antibiotic resistance may not have reached an alarming stage yet in these areas as compared to other regions of the world. Variation in sensitivity and resistance pattern may be partly due to different isolation sources and environmental conditions as well^39^. However, several antibiotics used to characterize the bacteria in our study, except florfenicol, erythromycin and sulphonamide group, are prohibited for use in aquaculture^40^.

In conclusion, the isolation of *Aeromonas hydrophila* (AM-31) and *A. jandaei* (AM-05) from diseased *A. testudineus*, their role in pathogenicity by re-infection and the presence of virulent genes corroborates the active contribution of motile Aeromonads towards pathogenesis in cultured fish species. We confirm that *A. hydrophila* and *A. jandaei* can infect farmed *A. testudineus*, evident as visible ulcers and haemorrhage on the body and fins as well as tissues within. To the best of our knowledge, this is a first record on the mortality of farmed *Anabas* naturally-infected by *A. hydrophila* as well as *A. jandaei*, with no available records on pathogenicity of the latter bacterium in this fish. As culture of *A. testudineus* in India is gaining momentum given its high market demand, the present study will act as baseline data on the pathogenic role of *A. hydrophila* and *A. jandaei* in this fish, opening up future scopes for investigation on their possible transmission (or opportunistic role) onto other cultivable fish species of the region, and therefore, aid in the advancement of appropriate preventive measures and management activities.

## Methods

### Ethical statement

The protocols of the present study were reviewed and approved by the Institutional Animal Ethical Committee (IAEC) of Gauhati University, Assam, India (Permit No. IAEC/Per/2018/PP-IAEC/2018-039). All experiments were performed in accordance with the IAEC guidelines and regulations.

### Bacterial isolation

Live specimens (*N* = 50) of naturally-infected *Anabas testudineus*, exhibiting haemorrhagic ulcerations and tail-rot (see Supplementary Fig. S1 online), were collected from a local fish farm (26°27ʹ21″ N; 91°22ʹ53″ E) in Assam (India) between November 2018 and January 2019. The moribund *Anabas* individuals were euthanized using an overdose of clove oil prior-to-use for bacterial isolation and histopathological studies. After sedation, diseased fishes were aseptically washed with 70% ethyl alcohol and the ulcerated areas treated with a heated scalpel in order to reduce contamination. After sterilization, swabs were taken with a sterilized loop from the ulcerated body parts as well as kidneys and streaked on sterilized nutrient agar (NA) (Himedia) plates. The NA plates were then incubated at 29 (± 2) °C for 48 h and the growth of bacterial colonies (if any) was observed. Two dominant growing bacterial colonies were selected and purified through subculture by multiple streaking on NA medium. These were then marked and labelled as two isolates: AM-31 and AM-05. The isolates were further processed for biochemical and molecular identification. The bacterial isolates were also cultured in LB broth (Himedia) containing 10% glycerol and then kept in − 80 °C for long time storage.

### Morphological and biochemical characterization of bacterial isolates

Pure cultures of the AM-31 and AM-05 isolates were inoculated on NA medium overnight at 29 °C and the colony morphology was observed. The isolates were subjected to Gram staining followed by microscopic observation. Sub-culturing of both isolates in LB at 29 °C was done prior to biochemical characterization as per Martin-Carnahan et al.^42^. Probable species level identification of the bacterial isolates followed Popoff and Véron^43^ and Abbott et al.^44^.

### Molecular identification based on 16S rRNA and *rpo*B gene amplification

The genomic DNA of both isolates AM-31 and AM-05, cultured in LB at 29 °C overnight in an orbital shaking incubator, was extracted using QIAamp DNA Mini Kit (Qiagen, Germany) following the manufacturer’s protocol and stored at − 20 °C until use. The 16S rRNA gene was amplified using universal bacterial primers according to the standard protocol of Martin and Collen^45^ and the *rpo*B (RNA polymerase beta subunit) gene was amplified following the protocol of Liu et al.^46^, respectively. PCR reactions for each gene comprised of 25 µl ready-to-use master mix (R2523-100RXN, Sigma), 2.5 µl each of the forward and reverse primers and 5 µl DNA templates, and the final volume was made to 50 µl using nuclease-free water. A negative control (without DNA template) was taken as well. The qualities of the PCR-amplified products for both genes were checked by 1% (w/v) agarose gel (containing ethidium bromide) electrophoresis in TBE buffer. The PCR products were then purified using QIA quick Gel Extraction Kit (Qiagen, Germany) following the manufacturer’s protocol, and outsourced to AgriGenome Labs Private Limited, Cochin (India) for Sanger sequencing. Sequences obtained for the gene datasets were edited using Clustal W (inbuilt in MEGA 7)^47^ and a BLAST search was performed in the National Centre for Biotechnology Information (NCBI) database to identify their nearest neighbour(s). Phylogenetic trees were constructed from evolutionary distances using the unweighted pair group method with arithmetic mean (UPGMA) with Kimura 2-parameter (K2P) evolutionary sequence models. The tree branches were authenticated by bootstrap analyses of 1000 replicates. *Pseudomonas sharmana* (NR 043470.1) and *P. pectinilytica* (NR 156860.1) were taken as out-groups for rooting trees of the 16S rRNA dataset.

### Pathogenicity test

To test the pathogenicity of isolates AM-31 and AM-05, disease-free, healthy individuals of *A. testudineus* (*N* = 160; weighing approximately 22–40 g) were collected from aquaculture farms of the Barpeta District of Assam, India, with a constant practice of carp, murrel, catfish and/or *Anabas* culture. After collection, the fishes were acclimatized in 80 L glass aquaria (@ 30 individuals in each aquaria) 14 days prior-to-challenge. All disease-free individuals were anaesthetized in a 50 ppm clove oil solution following Shameena et al.^41^, with a recovery time of 4–5 min, prior to proceeding with the experimental infections.

AM-31 and AM-05 isolates were cultured in LB at 30 °C overnight the day before the experimental challenge with continuous shaking. The isolates were then centrifuged and suspended in PBS at six different concentrations of each as follows—for AM-31, the concentrations ranged from 3.4 × 10^2^–3.4 × 10^7^ CFU/fish; while for AM-05, the concentrations ranged from 4.2 × 10^2^–4.2 × 10^7^ CFU/fish, respectively (CFU values were calculated from conventional plate counting) (Table 2).

An experimental setup was designed comprising 12 groups. Each group was represented by 10 individuals of *A. testudineus* in a 30 L aquarium. Of these, six groups were separated for the bacterial isolate AM-31, and 0.1 ml of each of the six prepared AM-31 bacterial suspensions was injected intraperitoneally to the individuals of each group in order of their increasing concentrations. The same experiment was conducted for the remaining six groups with the prepared suspensions of the bacterial isolate AM-05.

Also, two groups were prepared as control against the bacterial isolate AM-31, both comprising of 10 individuals of *A. testudineus* in 30 L aquaria. Of these, one group was injected with 0.1 ml of PBS to each individual; while the other group was maintained as without any injection. Similarly, two additional groups (each with 10 individuals) were prepared as control against the isolate AM-05.

Clinical signs and mortality (if any) of fish in all the respective groups were then recorded everyday up to 15 days post challenge.

### Haemolysis assay

Haemolysis test was carried out in tryptic soy agar plates (TSA) with 5% whole sheep blood^48^. Both the bacterial isolates were streaked on TSA containing 5% sheep RBCs, incubated at 30 °C for 24 h and checked for haemolytic activity by detecting a lucid haemolytic zone formed around the colony. The zone diameters were measured with a plastic measuring ruler.

### Detection of virulence-associated genes

PCR amplification of the virulence-associated genes [aerolysin (aerA) and enterotoxin (act)] from the genomic DNA was performed using different set of primers, amplification conditions and the expected product sizes (Table 4).

**Table 4:** Primers used for PCR detection of virulent genes in two bacterial isolates (AM-31 and AM-05) from naturally-infected *Anabas testudineus* (*Aerolysin, ^#^Enterolysin).

Target gene	Primer sequence (5ʹ → 3ʹ)	Size of PCR amplicon (bp)	Annealing temperature (°C)	References
aerA*	CAAGAACAAGTTCAAGTGGCCA ACGAAGGTGTGGTTCCAGT	309	59	Wang et al.^55^
Act^#^	GAGAAGGTGACCACCAAGAACA AACTGACATCGGCCTTGAACTC	232	66	Kingombe et al.^56^

### Histopathological study

Kidney and liver tissues from *Anabas* post artificial infection with the bacterial isolates (@ dozes of 3.4 × 10^6^ CFU/fish for AM-31 and 4.2 × 10^6^ CFU/fish for AM-05; each with 100% morality) were fixed in 10% neutral buffer formalin (NBF) for histological studies. Tissues were then subjected to dehydration through a series of increasing alcohol grades, cleared with xylene and embedded in paraffin following standard method. The paraffin-embedded tissues were sectioned at 5 µm using a microtome and stained with haematoxylin and eosin^49^. Pathological changes manifested in the tissue sections were examined under a Leica DM 3000 microscope at 40 × magnification.

### Antimicrobial susceptibility test

Antibiotic sensitivity or resistivity was tested on Mueller–Hinton agar plates inoculated with 0.1 ml of overnight broth culture of both isolates (1 × 10^7^ CFU/ml of AM-31 and 1 × 10^6^ CFU/ml of AM-05) following Bauer’s disc diffusion method^50^. Antibiotic-impregnated discs (Himedia, India), comprising of 20 antimicrobials, were placed on the solid medium and the plates were incubated at 30 °C. After 24 h, the antibiotic sensitivity was determined by measuring the diameter (in mm) of the zone of inhibition formed around the disc, which were expressed as mean ± standard deviation of five replicates. The radius of zone of inhibition was scaled from the centre of the antibiotic disc to the end of the clear zone where bacteria could be seen growing. Results were interpreted on the basis of zone diameter as sensitive and resistant following Clinical and Laboratory Standards Institute^51^.

### Statistical analysis

The mortality data obtained were subjected to Probit Analysis following Finney^52^ for determination of LC_50_ values using SPSS (Version 16)^53^ and Minitab (Version 19.2.0)^54^ software.

## Supplementary Information

Supplementary Figure S1.

## Data Availability

All data generated during the current study are included in this article and its Supplementary Information files. The nucleotide sequences are freely accessible at the NCBI database (Accession numbers: MN097841, MN204041, MN977195 and MN977194).
