# Supplementary material for: Isolation and characterization of two virulent Aeromonads associated with haemorrhagic septicaemia and tail-rot disease in farmed climbing perch Anabas testudineus
Source: Sci Rep. 2021 Mar 12;11:5826. doi: 10.1038/s41598-021-84997-x (PMC7971006; doi:10.1038/s41598-021-84997-x)

Isolation and characterization of two virulent Aeromonads associated with haemorrhagic septicaemia and tail-rot disease in farmed climbing perch *Anabas testudineus*

Abhishek Mazumder, Hrishikesh Choudhury, Abhinit Dey & Dandadhar Sarma

Supplementary Information

Figure S1 Naturally-infected *Anabas testudineus* showing ulcerations (U) and tail-rot (TR) collected from a fish farm in Assam, northeast India.

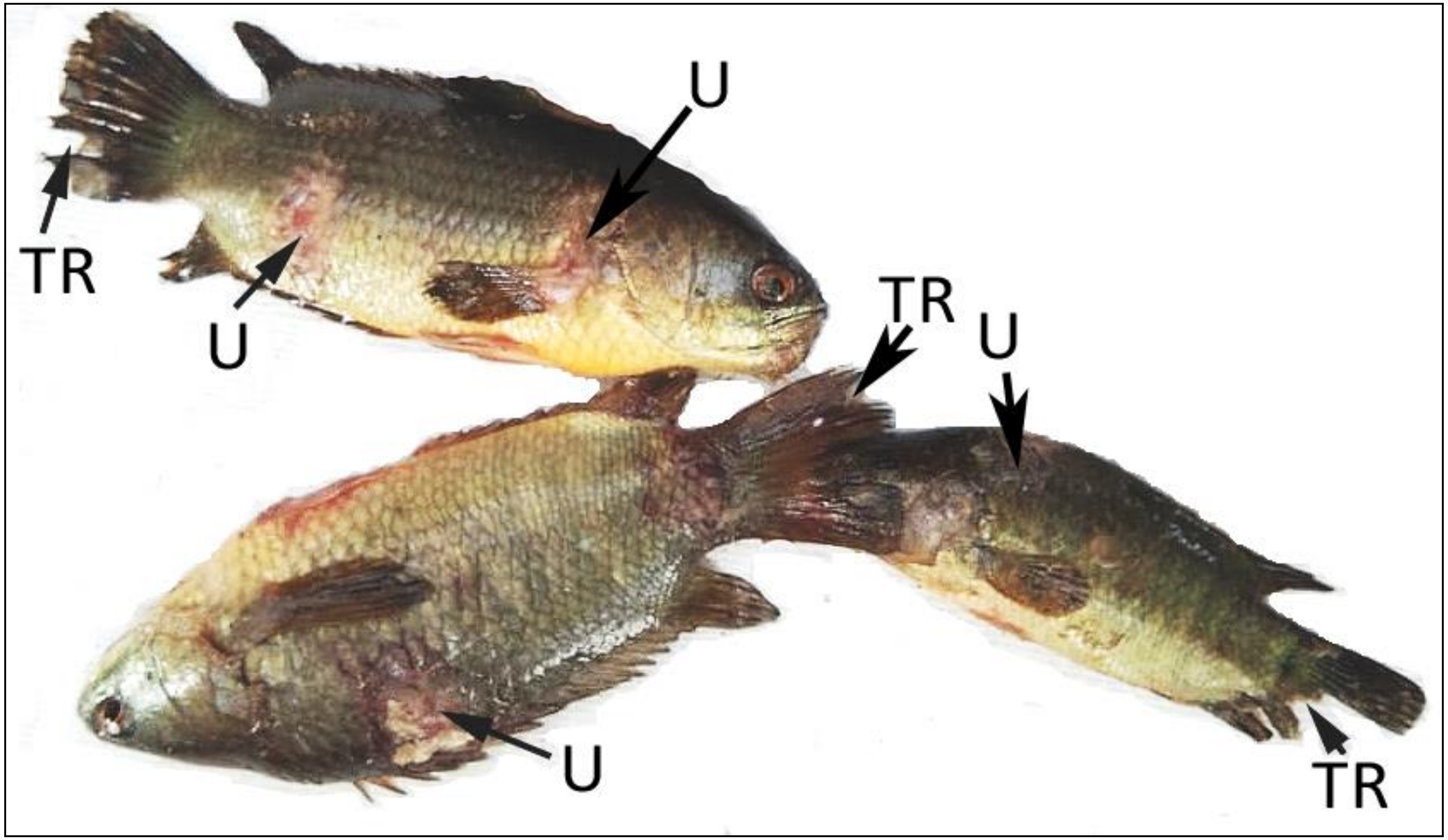

Supplement: Supplementary file 1 — Supplementary Figure S1. [file 41598_2021_84997_MOESM1_ESM.pdf]
